# Experimental Research on Fatigue Crack Growth Behavior of Diffusion-Bonded Titanium Alloy Laminates with Preset Unbonded Areas

**DOI:** 10.3390/ma15155224

**Published:** 2022-07-28

**Authors:** Yang Liu, Shutian Liu

**Affiliations:** 1 College of Transportation Engineering, Dalian Maritime University, Dalian 116026, China; liuyang1042@dlmu.edu.cn; 2 State Key Laboratory of Structural Analysis for Industrial Equipment, Department of Engineering Mechanics, Dalian University of Technology, Dalian 116024, China

**Keywords:** diffusion-bonded titanium alloy laminates, preset unbonded area, fatigue behavior, crack growth, toughening design

## Abstract

This paper aimed to study the fatigue crack growth behavior of diffusion-bonded titanium alloy laminates (DB-TAL) with preset unbonded areas using an experimental method to understand the toughening mechanisms of presetting unbonded areas in DB-TAL. For two series of specimens of DB-TAL with preset unbonded areas with an open hole, which have a pre-notch at the open hole edge, fatigue experiments under tension–tension cyclic loading were conducted. The fatigue crack growth process, the crack growth rate, and the stress intensity factor on the crack front were analyzed. The results showed that the preset unbonded area leads the crack away from the stress concentration zone and slows down the crack growth rate. Therefore, the preset unbonded area significantly improved the fracture property of DB-TAL.

## 1. Introduction

Titanium alloy has been widely used in the manufacture of key structural parts for aircraft due to its high specific strength, excellent corrosion resistance, and thermal stability [1,2,3,4]. Although they have excellent structural performance, titanium alloys have low damage tolerance performance [5,6,7,8] and are susceptible to initiated cracks, i.e., their residual crack growth life after a crack occurs is very short. Therefore, toughening the design of titanium alloy structures is an important topic.

The main aim of toughening the design of titanium alloy structures is to prolong the crack growth life. The so-called crack growth control mechanism is used to prolong the crack growth life. In such a case, a local stiffness mutation is introduced to the structure, e.g., stiffening a rib on the panel [9], arranging bonded crack retarders on the crack [10], making crenellations between the stringers [11], and so on. Nowadays, a type of diffusion-bonded laminate has been receiving much attention because of its good control of crack growth.

Diffusion bonding (DB) is a solid-state bonding technique without the presence of a liquid phase and is a commonly used process in machining titanium alloys [12,13,14,15]. Titanium alloy laminate is manufactured by the diffusion bonding of several thin titanium sheets under a specific time, applied pressure, and bonding temperature. This enables one to take the thickness and number of layers of a diffusion-bonded plate as the design parameters, which are more flexible to different structural demands.

Based on the above characteristics, Reference [16] proposed a type of damage tolerance improving method based on DB-TAL. The mask solder was designedly set between the sheets to prevent diffusion bonding so the formed DB-TAL contains the locations unbonded in the interfaces. The diffusion-bonded titanium alloy laminates with preset unbonded areas (DBTALPUA) have a little decline in static strength under in-plane loading [17], and researchers have carried out a lot of work on DBTALPUA. Reference [18] built the numerical analysis model for the fatigue failure process of DBTALPUA and obtained the unbonded area effect law for the crack growth life of the laminates in a parameter analysis. Reference [19] obtained the process of fatigue failure of DBTALPUA with surface cracks. The results showed that an unbonded area can change the crack growth path of a surface crack and prolong the crack growth life of a laminate. Reference [20] analyzed joints made of DBTALPUA using a numerical method. The results showed that the damage tolerance of the joints significantly improved.

The research mentioned above showed that DBTALPUA has enormous potential in damage tolerance performance, especially when used in structures with stress concentration (e.g., structure with an open hole or joint) under in-plane loading. Crack surface analysis based on the experiments is an important method for understanding the failure process. At present, there is a lack of experimental research on DBTALPUA related to stress concentrations. Therefore, in this paper, experiments were conducted on the laminates. Through the specimens’ crack surfaces in the experiments, the crack growth behavior was studied. The conclusion could provide a foundation for the optimal design of DBTALPUA.

## 2. Experimental Details

### 2.1. Specimens

An alpha–beta general-purpose titanium alloy Ti-6AL-4V was used for this study. The composition is shown in Table 1.

The production of the DBTALPUA specimens included the following steps.

**Figure 1:**
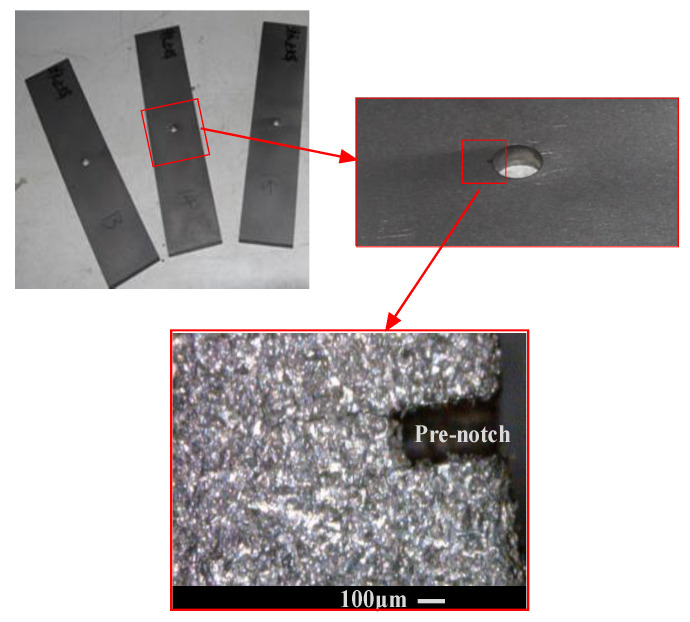
The pre-notch shape and location.

1. Ti-6Al-4V titanium alloy sheets with 2 mm thickness were chosen to manufacture the laminates.
2. The solder resistance was designedly set between the sheets before the diffusion-bonding process for producing the unbonded areas at the interfaces.
3. Four titanium alloy sheets were bonded using diffusion-bonding technology at 900 °C/1.5 MPa/1.5 h.
4. The laminates were cut into specimens following the design. Then, a through-thickness bolt hole was drilled at the center of each specimen. The notch at the single hole edge was made by electro-discharge machining, as shown in Figure 1.

**Figure 2:**
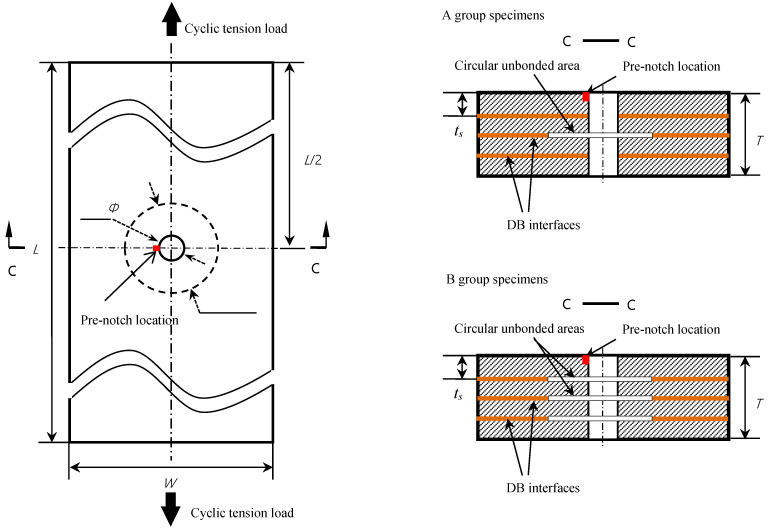
The layout of Groups A and B DBTALPUA specimens.

The specimens had a length of *L* = 180 mm, a width of *W* = 40 mm, and a thickness of *T* = 8 mm. The layout of the specimens is shown in Figure 2.

### 2.2. Test Conditions and Procedures

**Figure 3:**
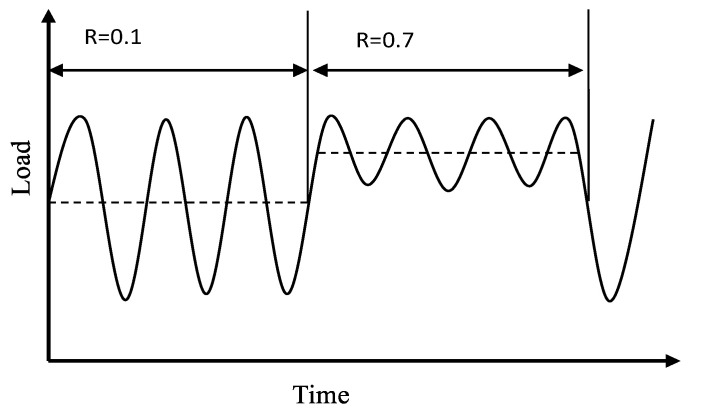
The loading spectrum.

The test was carried out using mts-810 1000 kN fatigue test machine at room temperature. This research focuses on the fatigue crack growth behavior of the laminates. So, the pre-crack was made by cyclic loading from the pre-notch location. A digital microscope was used to observe the position of the root of the notch until the crack grew to 2 mm. The benchmarking method was used to study the crack growth in the laminates. During the test, two kinds of stress ratios (*R* = 0.1 with a frequency of 8 Hz and *R* = 0.7 with a frequency of 20 Hz) were used to alternately load the specimens until they fractured. TestStarIIs control system was employed to program the load spectrum, which is shown in Figure 3. The loading part *R* = 0.1 is the formal load and the loading part *R* = 0.7 is the marker load. The maximum tensile load of the tensile–tensile cyclic loading was 73.6 kN, corresponding to the average peak stress of 230 MPa in the specimen, and the average peak stress of the central section containing the holes was 270 MPa. The details are listed in Table 2.

## 3. Experimental Results

### 3.1. The Fracture Morphology Analysis of the Crack Propagation Process

The beach marking method was employed in the experiment. The “beach marking” dividing line forms on the fracture section of the specimen. The line can reflect the crack front shape and location when the identified *R* = 0.7 is loaded. By analyzing these dividing lines, the position and shape of the fatigue crack front in the crack growth process can be obtained.

#### 3.1.1. The Specimens with One Unbonded Area

**Figure 4:**
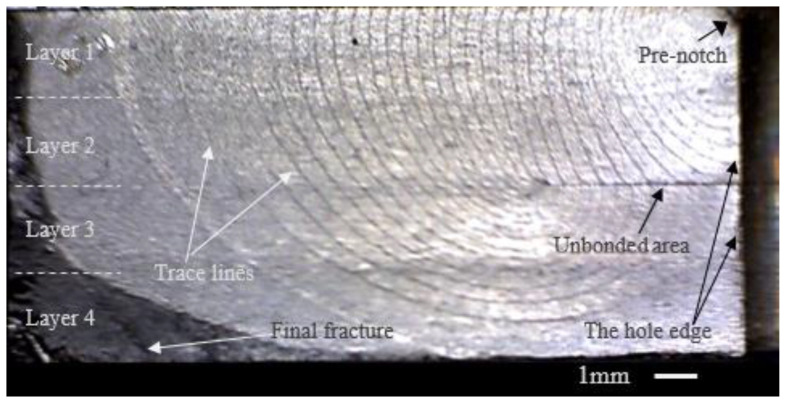
The base information of the fracture surface of Specimen A-1 in Group A.

Figure 4 shows the fracture surface of specimen one. Note that in this paper, the crack is on a single edge of the hole. The other edge suffered an instantaneous fracture when the fatigue crack’s edge fractured. This paper focuses on the fatigue crack growth process, so the edge with the fatigue crack growth was shown as each specimen’s fracture surface. Most of the fracture surface is flat because of the uniaxial tension–tension fatigue loading. However, in the final fracture location, the fracture surface is uneven, which is indicated in Figure 4 in dark gray. The features of the fracture surface are indicated by the white font in Figure 4. The specimen was made with four layers of 2 mm titanium alloy sheets using diffusion-bonding technology. The unbonded area was set in the interface between layer 2 and layer 3, which is seen as a dark segment on the fracture surface. The unbonded area size in each specimen is different since the preparation techniques for the titanium alloy laminates with the unbonded areas are not mature enough at present. The lengths of the unbonded areas on the fracture surface in these four specimens are shown in Table 3. An important feature on the fracture surface is the beach marking lines. The lines can reflect the location and shape of the crack front during the crack growth process. Here we call these lines trace lines. The same cycles were experienced between the neighboring trace lines. Therefore, the density degree of the trace lines can give the crack growth rate determination on the nature of the fracture surface. If the trace lines have small spaces between them, it means the crack grew slowly, and large spaces between the lines mean a fast crack growth rate.

From the trace lines on the fracture surface in Figure 4, the crack growth path was easily determined. The crack grew from the pre-notch on the hole edge at the upper-right corner in Figure 4. The crack front gradually formed an approximate quarter ellipse and expanded along the front. When the crack grew to the unbonded area, the upper half of the specimen was penetrated. However, the crack cannot expand deeper into the specimen at this moment because of the unbonded area. It grew along the unbonded area to the unbonded area boundary, which is shown as a point on the left end of the unbonded area in Figure 4. When the crack grew to the unbonded area boundary, the bottom of the crack front began to bypass the unbonded area. Notably, although the crack bypassed the unbonded area, the bottom of the crack front near the unbonded area boundary is relatively intensive. There are more and more differences in intensity between the two ends of the crack front from the bypass time. This means that the crack growth rate along the depth of the specimen was inhibited even though the crack bypassed the unbonded area.

**Figure 5:**
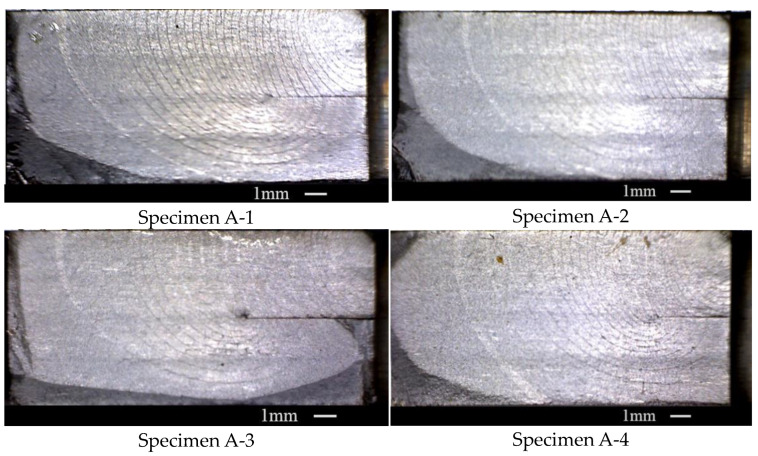
The four specimens’ fracture surfaces of Group A.

Four tests were conducted on the DB specimens with one unbonded area at the loading spectrum and the fracture surfaces are shown in Figure 5. The trends of the growth of the cracks in these four specimens are almost the same. The whole process can be divided into three stages by the different features of the crack growth. Stage one is the process of a crack growing from the pre-notch to the unbonded area. In this stage, the crack front has not yet touched the unbonded area and the unbonded area barely affected the crack growth. Stage two is the process during which the crack grew from the end of stage one to the unbonded area boundary. In this stage, the crack was blocked from growing into the depth of the specimen by the unbonded area and the crack grew along the unbonded area. Stage three is the process of a crack growing from the end of stage two to the fracture of the specimen. In this stage, the crack bypassed the unbonded area gradually until the fracture. Table 3 shows the details of these four specimens.

#### 3.1.2. The Specimens with Multi-Unbonded Areas

**Figure 6:**
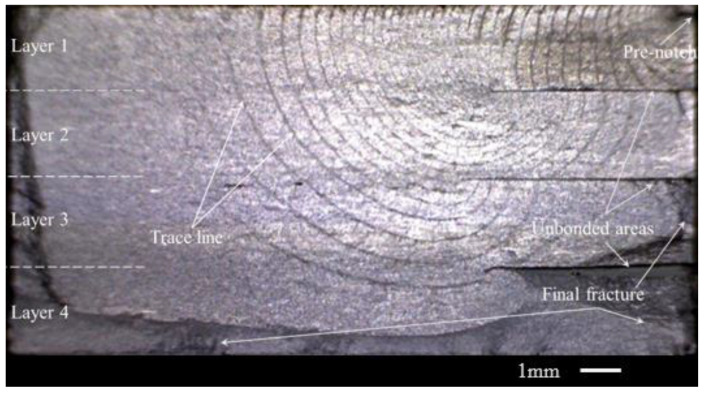
The base information of the fracture surface of Specimen B-1 in Group B.

Figure 6 shows the fracture surface of specimen one, which was set with three unbonded areas. This type of specimen was also made with four layers of 2 mm titanium alloy sheets using diffusion-bonding technology. The specimens were set with one unbonded area in each diffusion-bonding interface, which is different from the specimens with only one unbonded area. The loading stress level is the same with the specimens with only one unbonded area. However, in these tests, every loading period contains 1500 cycles *R* = 0.1 loading and 5000 cycles *R* = 0.7 loading. Therefore, in the fracture surface seen in Figure 6, 1500 cycles of *R* = 0.1 loading between the two adjacent trace lines were experienced. There are large differences in the density of the trace lines around the three unbonded areas. The trace lines around the upper unbonded area boundary are very intensive. In the locations of the other unbonded area boundaries, the trace lines are relatively sparse. This means that the first unbonded area that the crack goes through is the most effective for retarding the crack growth. When the crack grew to the second unbonded area, the fracture surface was too large so the second and the third unbonded areas have little effect.

If the crack growth process in the specimen with three unbonded areas was divided into three stages as described in the previous section, the first two stages would be similar to the crack growth in the specimens with one unbonded area. In the third stage in Figure 6, there is a notable phenomenon near the first unbonded area boundary. The trace lines in the third early stage are very intensive. The density of the trace lines in this location is much higher than that in the second crack growth stage. This variation in the trace line density is not obvious in the specimens with one unbonded area. The intensive trace lines near this location mean the fine inhibition in the crack growth of the unbonded area.

**Figure 7:**
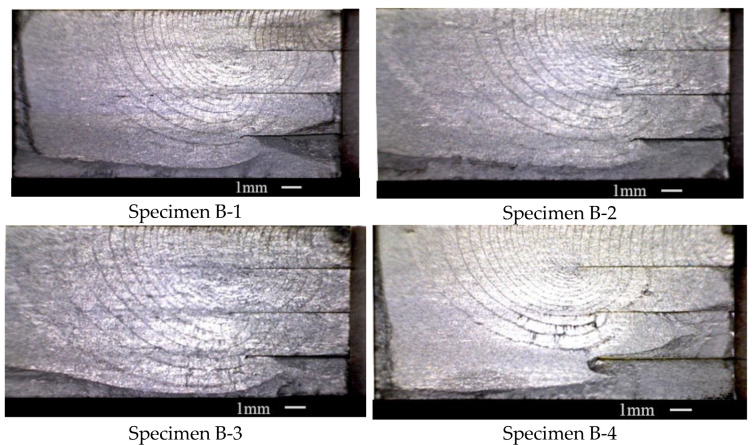
The four specimens’ fracture surfaces of Group B.

Four tests were conducted on the DB specimens with three unbonded areas at the loading spectrum, and the fracture surfaces are shown in Figure 7. The trace line layouts in each fracture surface are almost the same. Especially in the first unbonded area boundary location, the density of the trace lines on each fracture surface varied significantly. It can be seen that in the first unbonded area that the crack cross-by played a leading role in inhibiting crack growth.

### 3.2. The Fatigue Crack Growth Rate Analysis

**Figure 8:**
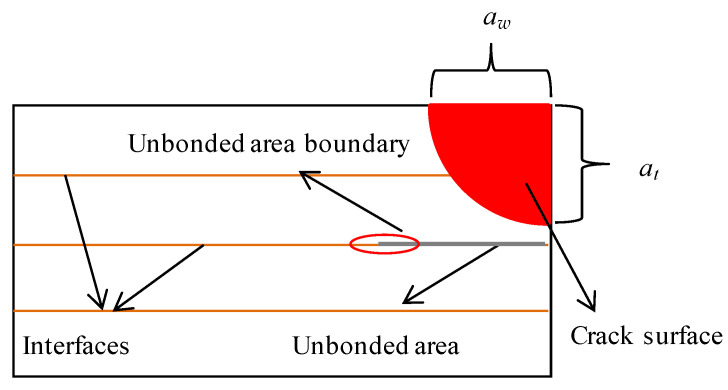
Section diagram of unbonded titanium alloy laminate.

In this section, the FCG (fatigue crack growth) rate is employed to describe the crack growth process in the laminates. The crack front is the curve in this 3D crack growth problem. For describing the crack length, two length parameters were adopted shown in Figure 8. One is *a_w_*, which represents the distance from the pre-crack on the hole edge to the point located on the specimen’s upper surface of the crack front curve. The other is *a_t_*, which represents the vertical distance from the specimen’s upper surface to the lowest point of the crack front curve. Due to the application of the beach marking method, the fracture surface of the specimen contains clear bands that represent the crack front position at that time. The two crack length parameters can easily be measured from the bands on the fracture surface. Moreover, the bands also contain the cycle time information. The FCG rate can be calculated according to the secant method in Formula (1).

(1) $${\left(\frac{da}{dN}\right)}_{i}=\left\{ \begin{array}{ll} \frac{a_{i+1}-a_{i}}{N_{i+1}-N_{i}} & i=1\\ \frac{1}{2}\left(\frac{a_{i+1}-a_{i}}{N_{i+1}-N_{i}}+\frac{a_{i}-a_{i-1}}{N_{i}-N_{i-1}}\right) & 1<i<n\\ \frac{a_{i}-a_{i-1}}{N_{i}-N_{i-1}} & i=n \end{array}\right.$$

The parameter *a* is the crack length; here we use *a_w_* and *a_t_* to describe the two aspects of the crack length. The parameter *N* is the total number of cycles corresponding to the trace line. The subscript *i* is the trace line number. The parameter *n* is the number of total trace lines.

**Figure 9:**
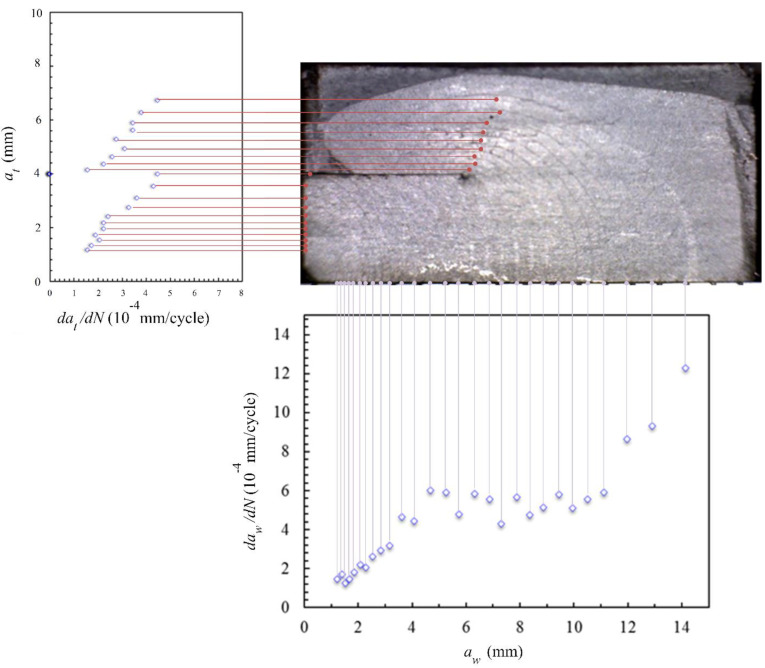
Variation in FCG rate with crack length *a_w_* and *a_t_* for specimen 1.

Figure 9 shows the crack growth rate varying with the trace line location, and Figure 9 further clarifies how we defined *a_w_* and *a_t_* in this paper. The density of the horizontal and vertical auxiliary lines also reflects the crack growth rate.

#### 3.2.1. The FCG Rate Analysis of the Specimens with One Unbonded Area

**Figure 10:**
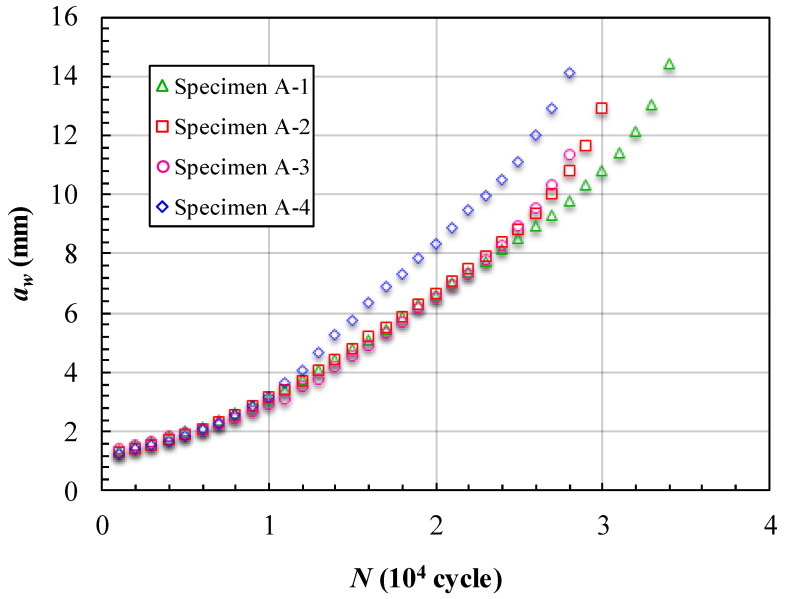
Crack length *a_w_* vs loading cycles *N* of specimens of Group A.

**Figure 11:**
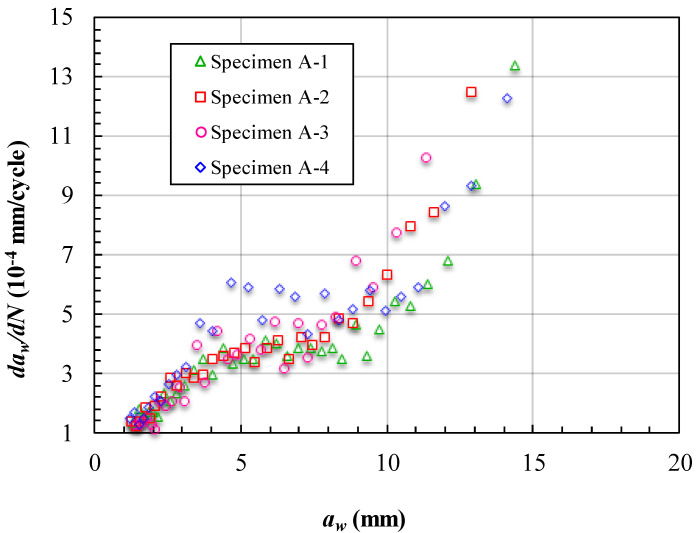
FCG rate at different crack lengths *a_w_* of specimens of Group A.

The fatigue crack growth data obtained for the cracks reflected on the surface (*a_w_*) of the specimens are presented in Figure 10 and Figure 11. Figure 10 shows the measured variations in the crack length and the corresponding number of cycles endured under the fatigue loading. Note that in Figure 10 the data point is measured from the trace line on the fracture surface. When the crack growth process reaches the final stage (near the instantaneous fracture zone), the crack growth rate is relatively fast. In addition, in this stage, the marker loading cannot leave a clear trace line on the fracture surface. Therefore, in Figure 10, there are differences between the final crack lengths in the specimens. The crack growth lives of the specimens in Figure 10 are about 30,000 cycles. Among them, the fourth specimen has the longest life and the first specimen has the shortest life. Before *a_w_* < 4 mm, the four curves are almost overlapping. This is because in this stage, the crack has not yet grown to the unbonded area boundary. However, after *a_w_* > 4 mm, the curve of specimen one is separate from the others. In Table 3 we can see that the first unbonded area in specimen one is much larger than the others. This is the main reason the curve of the specimen is separate. When the curves seen in Figure 10 begin to separate, the slope of each curve is almost unchanged for a while. For further analysis, the variations in the crack length and the corresponding crack growth rate are calculated to follow Formula (1), as shown in Figure 11. Figure 11 shows that the FCG rate is almost unchanged from *a_w_* =4 mm to *a_w_* =9 mm in each specimen. This means that the unbonded area makes the FCG rate increase slowly in this location. Moreover, when the crack growth bypassed the unbonded area boundary, the FCG rate increased again without any unusual jump. The unbonded areas make the FCG rate curves have a platform feature.

**Figure 12:**
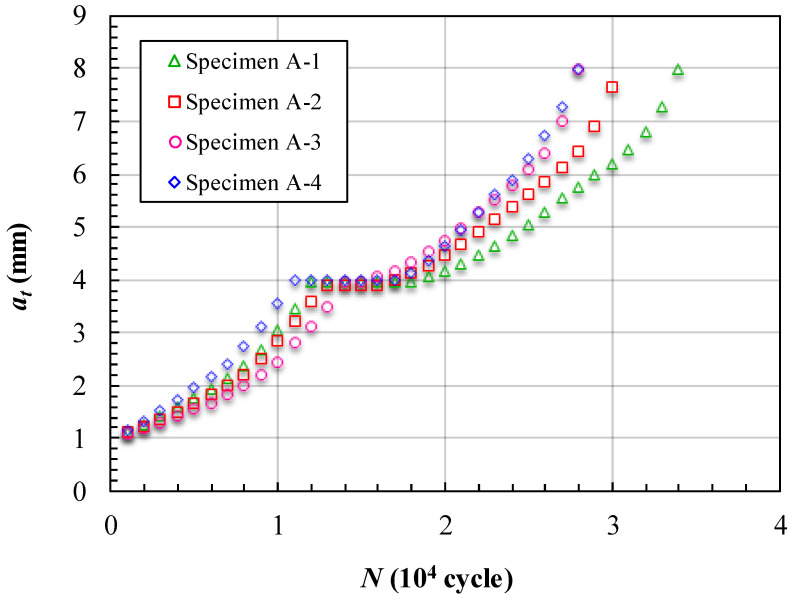
Crack length *a_t_* vs loading cycles *N* of specimens of Group A.

**Figure 13:**
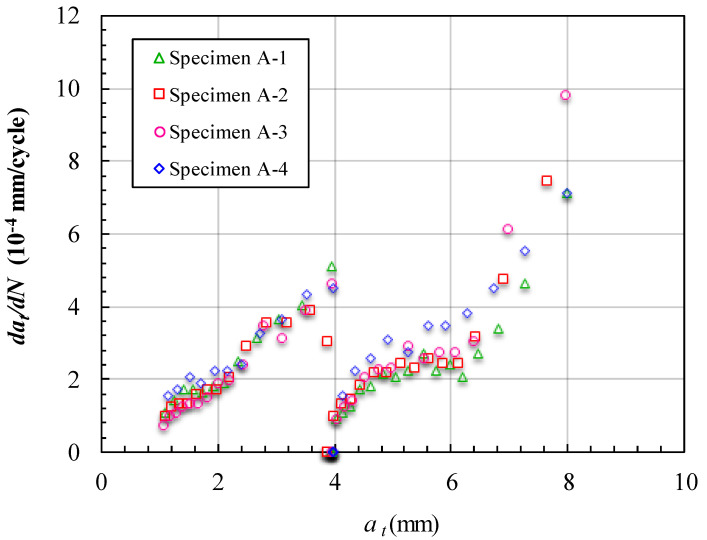
FCG rate at different crack lengths *a_t_* of specimens of Group A.

The fatigue crack growth data obtained for cracks reflected in the depth (*a_t_*) of the specimens are presented in Figure 12 and Figure 13. Figure 12 shows the measured variations in *a_t_* and the corresponding number of cycles endured under the fatigue loading. The trends for the four curves are almost the same and there is a remarkable feature of the curve in Figure 12. When *a_t_* reached 4 mm, the curves remained horizontal for a while and the corresponding cycles were from 12,000 to 18,000 cycles. This means that the crack did not grow along with the depth of the specimen in this horizontal segment loading time. In addition, the unbonded area was set at a depth of 4 mm in the specimen. This horizontal segment of the curve is stage two of the crack growth process described earlier. In this stage, the crack grew along the unbonded area and could not grow deeper under the effect of the unbonded area. Figure 13 shows the FCG rate varying with *a_t_*. There are sudden changes in the curves at the location *a_t_* =4 mm, which corresponds to the horizontal segment in Figure 12. Aside from this directly interdicting the crack growth along the depth, there was another indirect effect of inhibiting the crack growth because of the unbonded area. Although, when the crack began to bypass the unbonded area, *a_t_* began to increase with the cycle loading. The FCG rate at *a_t_* just past 4 mm was much less than that at *a_t_* close to 4 mm. This caused further delays in the crack penetrating the whole depth of the specimen.

#### 3.2.2. The FCG Rate Analysis of the Specimens with Multi-Unbonded Areas

**Figure 14:**
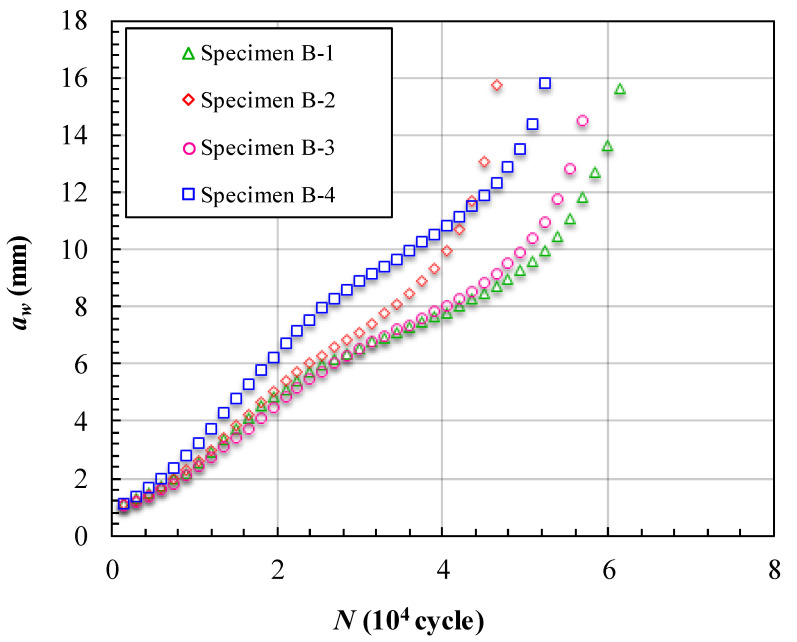
Crack length *a_w_* vs loading cycles *N* of specimens of Group B.

**Figure 15:**
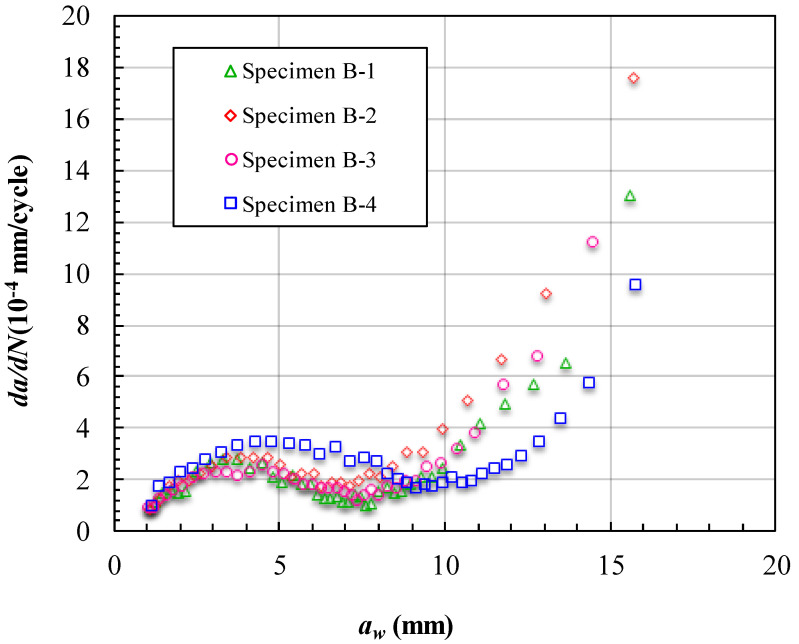
FCG rate at different crack lengths *a_w_* of specimens of Group B.

The fatigue crack growth data obtained for cracks reflected on the surface (*a_w_*) of the specimens with three unbonded areas are presented in Figure 14 and Figure 15. Figure 14 shows the measured variations in *a_w_* and the corresponding number of cycles endured under the fatigue loading. In these four specimens, specimen 7 experienced the most cycles at about 60,000 cycles. Furthermore, specimen 6 experienced the least number of cycles at about 45,000 cycles. The FCG lives of the specimens with three unbonded areas are much longer than those of the specimens with one unbonded area. The curves in Figure 14 have relatively large separation spaces. The separation spaces are mainly caused by the different sizes of the first unbonded area in the specimens. This means that when the first unbonded area was near the initial crack, the effect of the unbonded area was more sensitively influenced by the unbonded area size. Figure 15 shows the FCG rate varying with *a_w_*. There are obvious concave-down segments in the curves. This means that the FCG rate decreased along with the increase in *a_w_*. The concave-down segments are from approximately *a_w_* = 4 mm to 7 mm in the curves of specimens 5 to 7, and in the curve of specimen 8, the concave-down segment is from approximately *a_w_* = 5 mm to 9 mm. These concave-down segments are just the processes by which the crack fronts reached and bypassed the unbonded area boundaries. The size of the unbonded area has a large impact on the time that the concave-down segment appeared.

**Figure 16:**
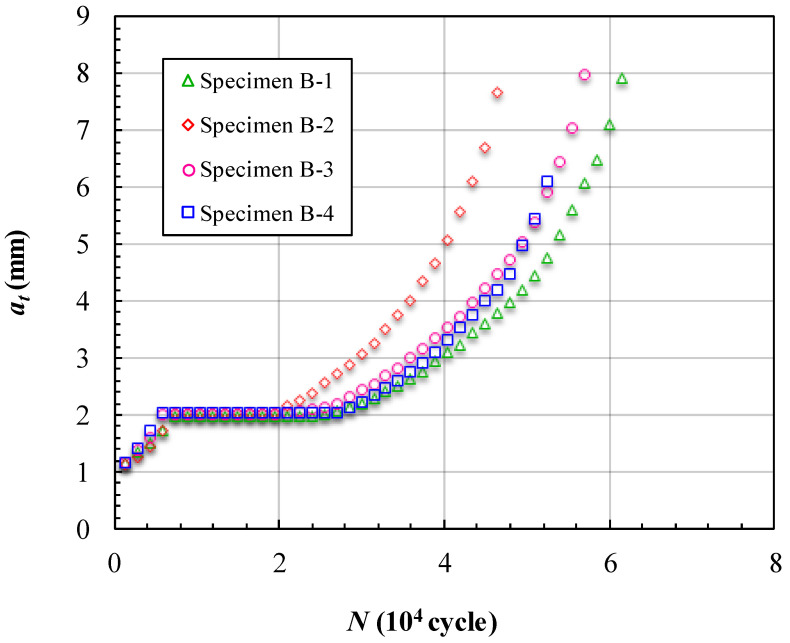
Crack length *a_t_* vs loading cycles *N* of specimens of Group B.

**Figure 17:**
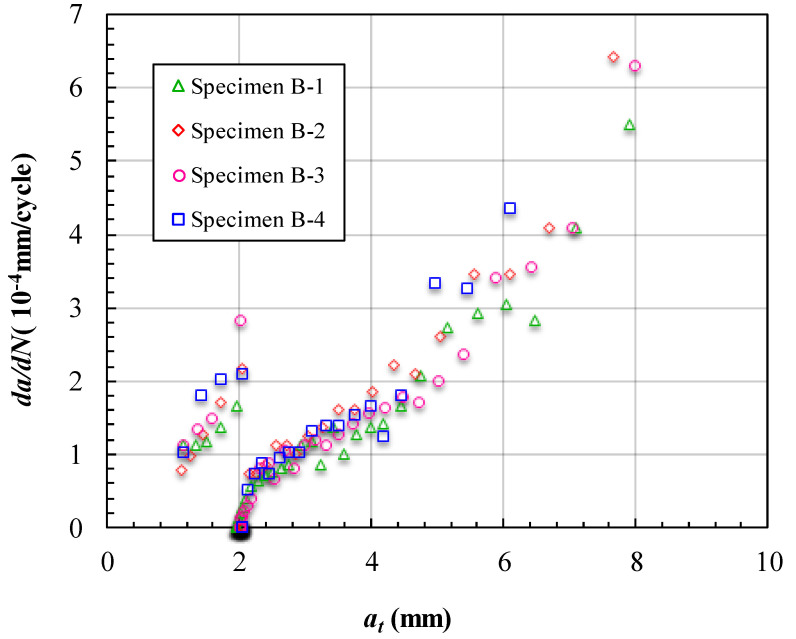
FCG rate at different crack lengths *a_t_* of specimens of Group B.

The fatigue crack growth data obtained for cracks reflected in the depth (*a_t_*) of the specimens with three unbonded areas are presented in Figure 16 and Figure 17. Figure 16 shows the measured variations in *a_t_* and the corresponding number of cycles endured under the fatigue loading. The horizontal segments of the curves in Figure 16 are located at *a_t_* = 2 mm, which is the location of the first unbonded area set. Although there was a total of three unbonded areas in each specimen, the curves have few abnormal features in the other two unbonded area locations. In Figure 16, we can see that specimen 6 bypassed the unbonded area first at about 20,000 cycles, and the other three specimens bypassed the first unbonded area at about 28,000 cycles. Notably, though the size of the first unbonded area in specimen 5 is much larger than in the other specimens, the cycles to bypass the first unbonded area approached specimens 7 and 8. This is because if the size of the first unbonded area is large, the failure area of the specimen is also large when the crack grew to the unbonded area boundary. The reason that specimen 6 took the shortest time to bypass the first unbonded area is due to the effects of stress concentration near the hole. In the layout design of the unbonded area in the structures with stress concentrations, it is better to coordinate the range of the stress concentration and the size of the unbonded area. Figure 17 shows the FCG rate varying with *a_t_* of the specimens with three unbonded areas. The trend is similar to that in the specimens with one unbonded area. The effect of the unbonded areas is mainly reflected in the first unbonded area, which can be seen from the curves in Figure 17. The curves have obvious gaps in the location *a_t_* = 2 mm and there are no obvious abnormalities in the locations of the other two unbonded areas.

### 3.3. The Damage Tolerance Analysis

**Figure 18:**
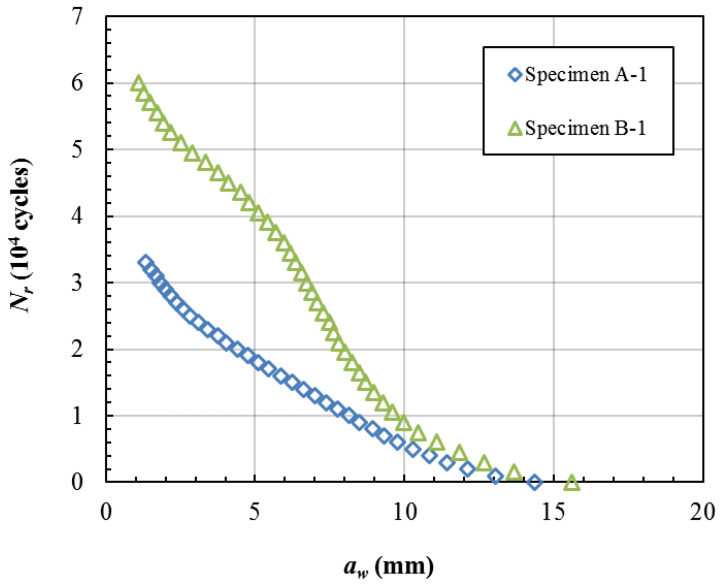
Crack length *a_w_* vs residual cycles *N_r_* of specimens.

A crack reflected on the surface (*a_w_*) is easily detected by checkout equipment. Figure 18 shows the residual cycles vs the crack length *a_w_* of Specimen A-1 and B-1. We assume that a 5 mm crack length can be detected easily, and in Figure 18 it can be seen that Specimen A-1 has about 18,000 residual cycles and Specimen B-1 has about 40,000 residual cycles when the crack lengths *a_w_* reach 4 mm. We assume that the repair cycles are 20,000 times and in Figure 18 it can be seen that the critical crack length *a_w_* is about 4 mm in Specimen A-1 and about 8 mm in Specimen B-1 when the residual cycles are about 20,000 times. From the above analysis, the damage tolerance of Group B is much higher than that of Group A. This is just the embodiment of the effect of the unbonded. When the crack length *a_w_* reaches a detectable length, the failure area is small and the crack front is far away from the region of the stress concentration in Group B. If there is no unbonded area in the laminates, the failure area is too large when the crack length *a_w_* reaches a detectable length. Therefore, the suitable distribution of unbonded areas can significantly improve the damage tolerance performance of the titanium alloy laminates.

## 4. The Stress Intensity Factor Analysis of Crack Fronts

In this section, we build the finite element model for the specimens to obtain the stress intensity factor *K* at the crack front and analyze the relationship between the stress intensity factor *K* and the crack growth rate.

### 4.1. The Numerical Model

**Figure 19:**
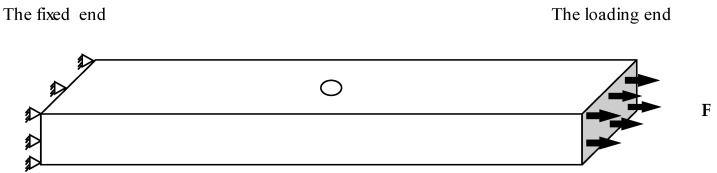
The geometry and boundary conditions of the specimen.

Figure 19 shows the geometry and boundary conditions of the model. The boundary conditions are as in the experiment, with one fixed end and one loading end.

**Figure 20:**
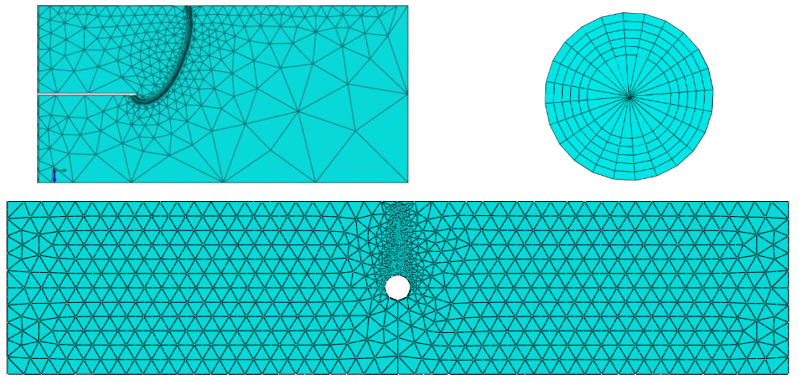
The finite element mesh of the entity and local crack in front of the model.

The finite element model was built based on ABAQUS 6.14. We built the models separately based on the trace lines from the experiments. Linear elastic analysis was employed to investigate the distribution of the stress intensity factors at the crack front. Young’s modulus was *E* = 110 GPa and Poisson’s ratio was *v* = 0.33. The main structure was represented by tetrahedron elements and the crack front location by hexahedral elements and wedge elements to simulate the stress singularity at the crack front. The J-contour integral method was employed to calculate the crack front information. For obtaining detailed information on the crack front, the mesh density at the crack front was very high with over 100 nodes in one crack front, as shown in Figure 20. For the mesh size sensitivity for the J-contour integral, we set 5 layers’ elements to calculate the J-contour integral values and adopted the mesh size to make the five J-contour integral values close to each other. The unbonded area was modeled by a geometry gap with a 20 μm width.

**Figure 21:**
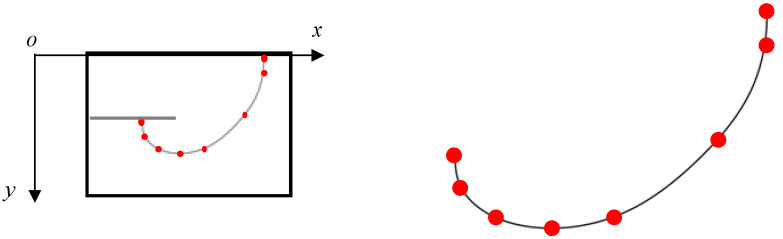
B spline modeling of trace line with 8 anchor points in the third stage.

The crack surface was defined by the seam method in ABAQUS, which has the same coordinates and separate nodes at the crack surface on both sides. The crack front shape was modeled by the B spline of the trace line and the anchor point number was adjusted by the trace line length shown in Figure 21.

### 4.2. Results Analysis

We chose one specimen of each type to calculate the stress intensity factor *K* at each crack front.

**Figure 22:**
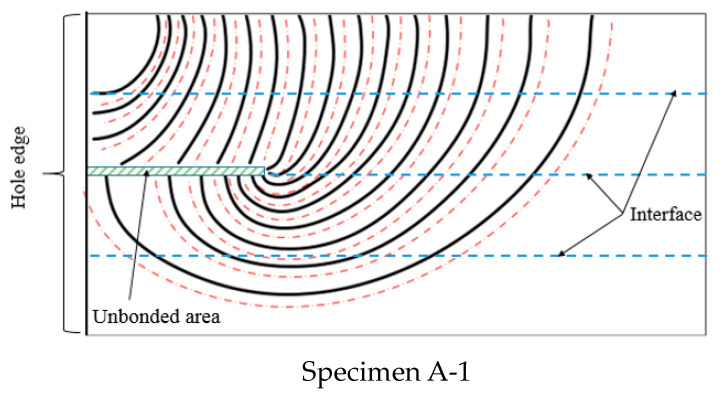

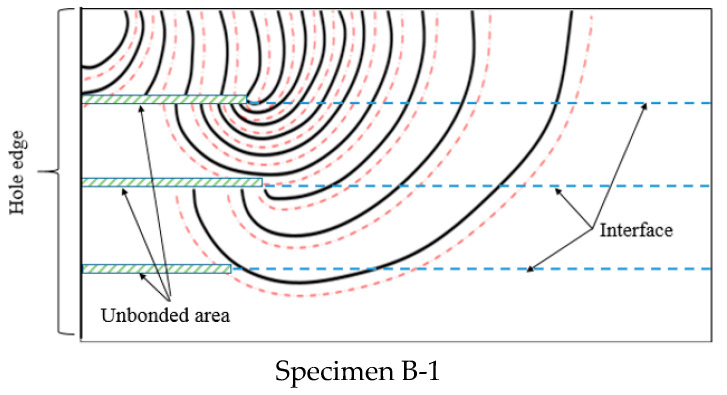
The stress intensity factor distribution at each crack front.

Figure 22 shows the trace line locations on the crack surface and the stress intensity factor distribution at each crack front. The distance between the dotted line and the trace line represents the normalized magnitude of the intensity factor. The stress intensity factor varied with the crack growth, as seen in Figure 22. We calculated all the trace line locations’ crack fronts, but for ease of visibility, the trace lines were plotted with one interval in Figure 22.

**Figure 23:**
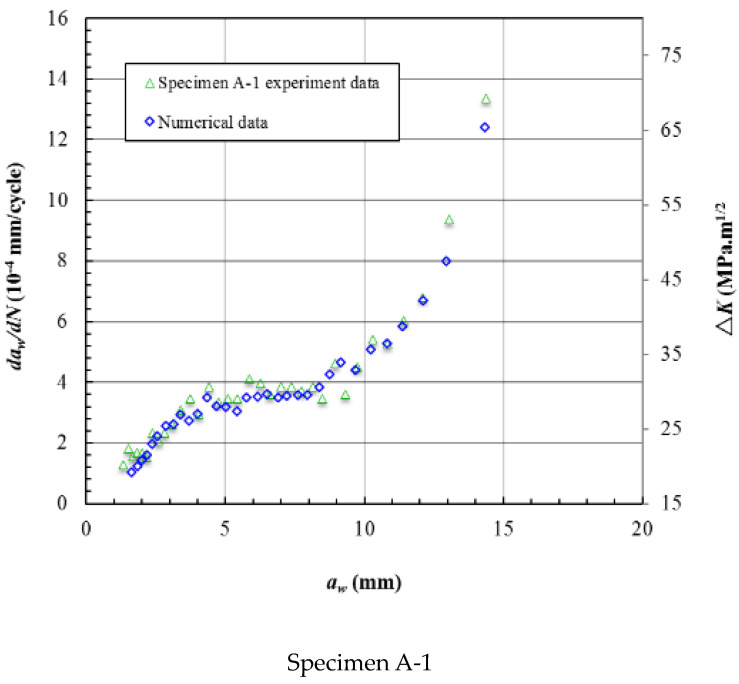

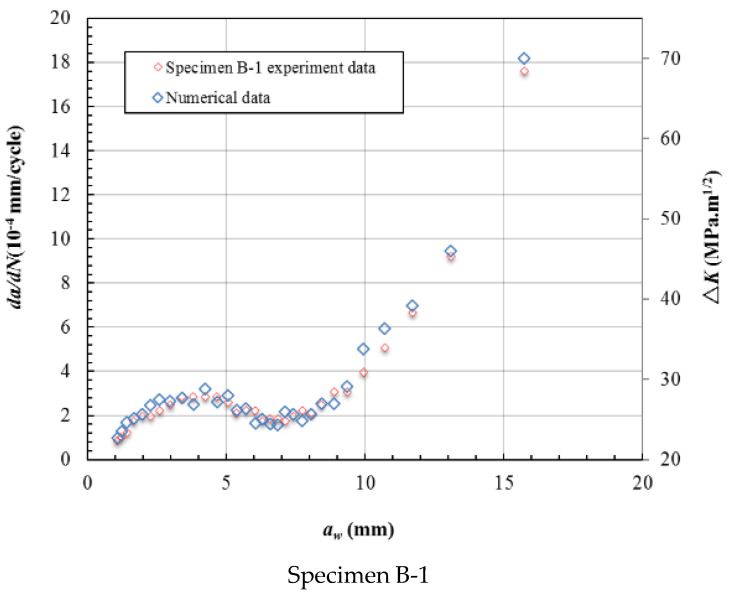
The crack growth rate and Δ*K* varying with the crack length *a_w_*.

**Figure 24:**
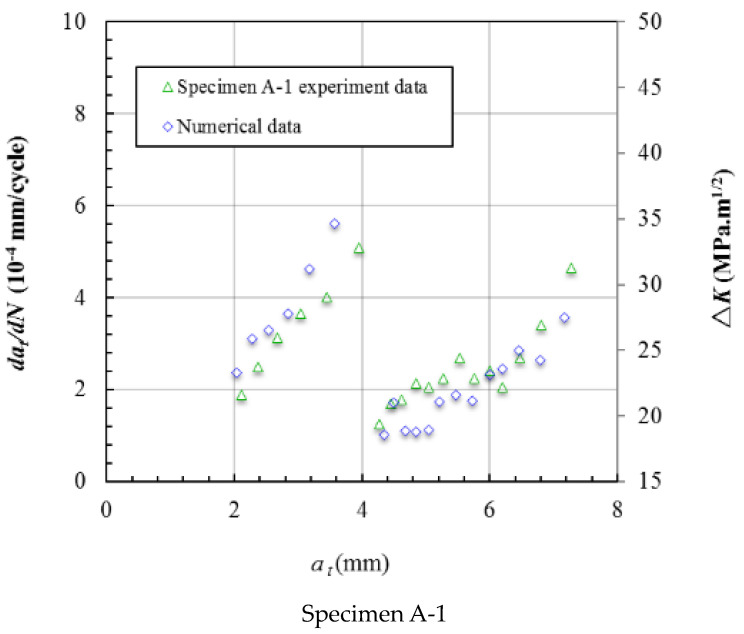

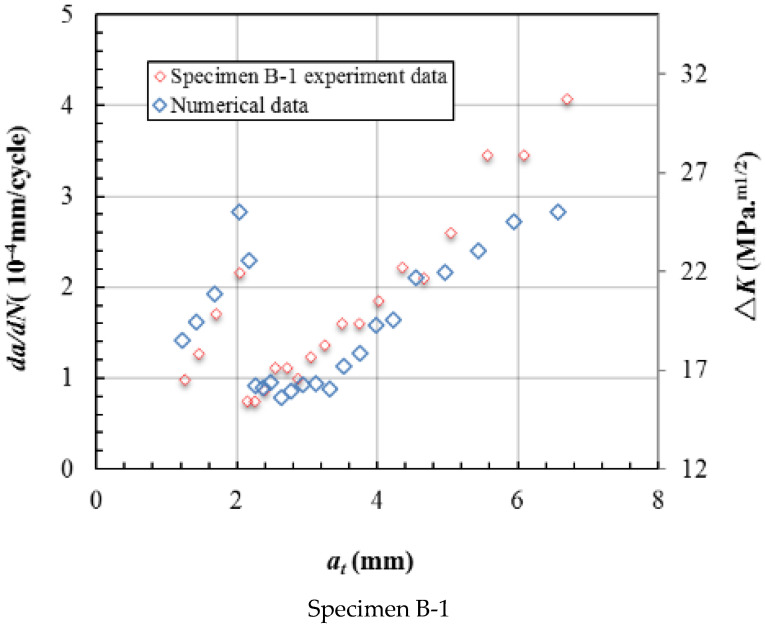
The crack growth rate and Δ*K* varying with the crack length *a_t_*.

The Δ*K* was calculated based on the *R* = 0.1 and the *K*_max_. Figure 23 gives the crack growth rate and Δ*K* varying with the crack length *a_w_*, and the Δ*K* data plotted on the secondary axis. The bounds of the axis can affect the shape of the data line but the trend will not be affected. Figure 23 shows that the crack growth rate data almost coincide with the Δ*K* data. This means that the crack growth rate is mainly affected by the stress intensity factor at the crack front. Figure 24 shows the crack growth rate and Δ*K* varying with the crack length *a_t_*, and the trends are also coincident.

**Figure 25:**
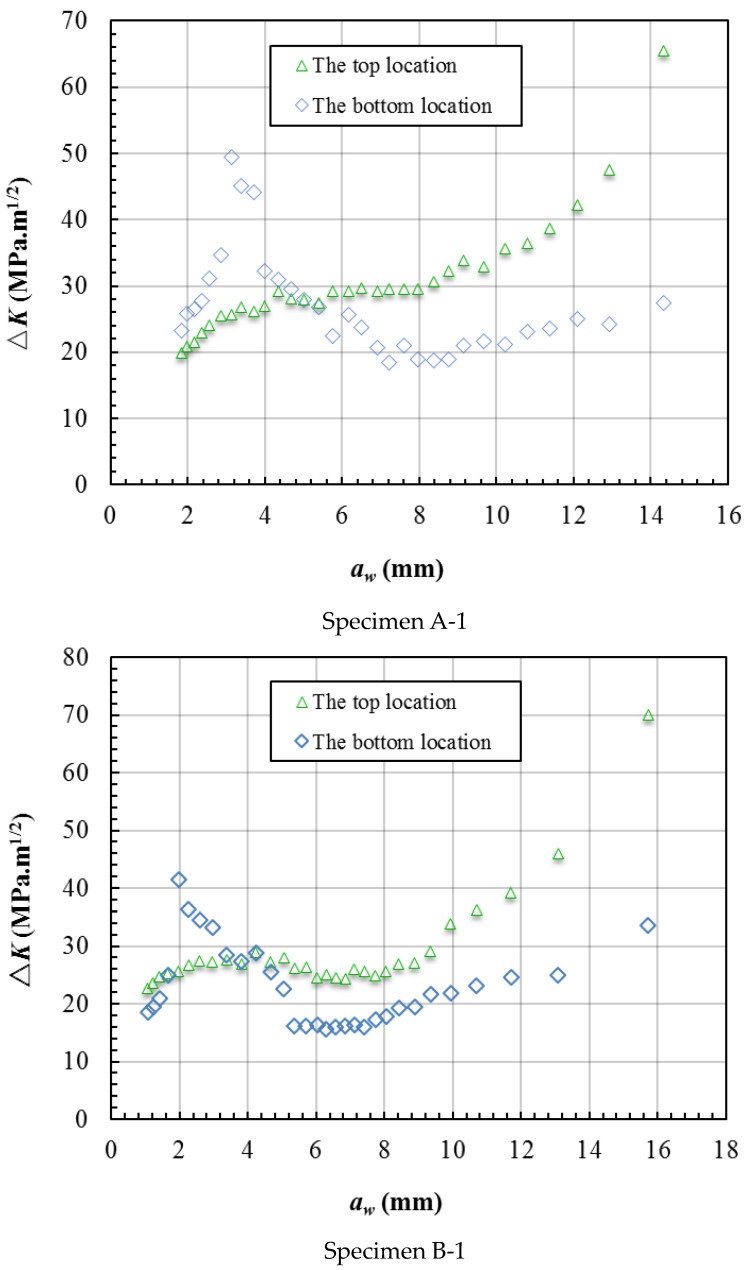
The Δ*K* at the top and bottom locations varies with the crack length *a_w_*.

Figure 25 shows the Δ*K* at the top and bottom locations in each crack front varying with the crack length *a_w_*. Figure 25 shows that the Δ*K* at the bottom is higher than that at the top before *a_w_* < 5 mm, mainly because the bottom of the crack front in this range is nearer to the hole edge where there is a high stress concentration. When *a_w_* > 7 mm, the bottom of the crack front reached the boundary of the unbonded area and the crack began to grow to the depth of the specimen. In this range, the Δ*K* at the bottom of the crack front is much lower than that at the top and there are two main reasons for this phenomenon. One reason is that the crack front had already grown far away from the hole edge and the degree of stress concentration had abated greatly. The other reason is that the shape of the crack front had less growth in the depth when the crack front was near the boundary of the unbonded area.

## 5. Summary

In this paper, eight specimens of two types of titanium laminates with unbonded areas were experimentally analyzed. Numerical models of the two types of specimens were built. Through the experiment and numerical data, several conclusions can be given as follows:

1. For the specimens that contained open holes, the unbonded areas significantly prolonged the fatigue growth life. This is because the unbonded areas can lead the crack far away from the hole edge where there is a high stress concentration. The fatigue crack growth life of the specimens in Group B was much longer than that of the specimens in Group A. The main reason for this was that the cracks in the specimens in Group B reached the unbonded areas early, and when the cracks were led away from the high-stress-concentration locations, there was a smaller failure area. From the FCG rate analysis of the crack lengths *a_w_*, there was a platform stage in the FCG rate of Group A and a declining stage in the FCG rate of Group B when the crack reached the boundaries of the unbonded areas. The fact that the FCG rate decreased with the increase in the failure area indicated that it was significant that the unbonded area guided the crack away from the stress concentration zone.
2. From the damage tolerance analysis, the titanium laminates were shown to have good damage tolerance performance because when the crack length *a_w_* reached a detectable length, the failure area was small and the crack front was far away from the region of the stress concentration. The structure still had enough residual life. From the comparison of Group A and Group B, the suitable distribution of unbonded areas significantly improved the damage tolerance performance of the titanium alloy laminates.
3. The numerical model gave the Δ*K* distribution of each crack front. Through the comparison of the experimental data and the numerical data, it could be seen that the trend in the crack growth rate almost coincided with the Δ*K* data. This means that the crack growth rate was mainly affected by the stress intensity factor at the crack front.

## Figures and Tables

**Table 1:** The composition of Ti-6Al-4V material.

Element	Al	V	Fe	C	O	N	Ti
Content (%)	5.5–6.75	3.5–4.5	≤0.5	≤0.1	≤0.2	≤0.05	Bal.

**Table 2:** The type of the specimens and the loading information.

Group	Material	Total Layers	Unbonded Areas	Average Stress	*R* = 0.1	*R* = 0.7
A (A1–A4)	TC4	4	1	270 MPa	1000	3000
B (B1–B4)	TC4	4	3	270 MPa	1500	5000

**Table 3:** The unbonded size reflected on the fracture surface and total cycles.

Specimen No.	Length(mm)	Total Cycles	*R* = 0.1 Cycles	Average
A-1	4.46	139,698	34,000	29,858.25
A-2	3.97	123,243	30,000	
A-3	5.91	113,364	28,000	
A-4	3.43	112,433	27,433	
B-1	4.71	268,953	61,500	54,375
B-2	4.25	204,432	46,500	
B-3	4.88	251,836	57,000	
B-4	7.17	233,532	52,500	
